# Adapting mHealth Interventions (PrEPmate and DOT Diary) to Support PrEP Retention in Care and Adherence Among English and Spanish-Speaking Men Who Have Sex With Men and Transgender Women in the United States: Formative Work and Pilot Randomized Trial

**DOI:** 10.2196/54073

**Published:** 2024-03-27

**Authors:** Albert Y Liu, Cat-Dancing Alleyne, Susanne Doblecki-Lewis, Kimberly A Koester, Rafael Gonzalez, Janie Vinson, Hyman Scott, Susan Buchbinder, Thiago S Torres

**Affiliations:** 1 Bridge HIV San Francisco Department of Public Health San Francisco, CA United States; 2 Department of Medicine University of California, San Francisco San Francisco, CA United States; 3 Department of Medicine University of Miami Miller School of Medicine Miami, FL United States; 4 Instituto Nacional de Infectologia Evandro Chagas Fundação Oswaldo Cruz Rio de Janeiro Brazil

**Keywords:** preexposure prophylaxis, PrEP, Spanish-speaking, Latino, transgender, men who have sex with men, mobile health, mHealth, HIV prevention, HIV, technology, formative, development, mobile technology, mobile app, text-messaging, SMS, app, application, USA, United States, health equity, mHealth tool, tool, acceptability, self-management, pilot, support

## Abstract

**Background:**

A growing number of mobile health (mHealth) technologies are being developed to support HIV preexposure prophylaxis (PrEP) adherence and persistence; however, most tools have focused on men who have sex with men (MSM), and few are available in Spanish. To maximize the potential impact of these tools in reducing gender and racial/ethnic disparities and promoting health equity, mHealth tools tailored to Spanish-speaking people and transgender women are critically needed.

**Objective:**

The aim of this study is to adapt and tailor 2 mHealth technologies, PrEPmate and DOT Diary, to support daily PrEP adherence and persistence among Spanish-speaking MSM and English- and Spanish-speaking transgender women and to evaluate the feasibility and acceptability of these tools.

**Methods:**

PrEPmate, an interactive, bidirectional, text messaging intervention that promotes personalized communication between PrEP users and providers, and DOT Diary, a mobile app that promotes self-management of PrEP use and sexual health through an integrated electronic pill-taking and sexual activity diary, were previously developed for English-speaking MSM. We conducted 3 focus groups with 15 English- and Spanish-speaking transgender women and MSM in San Francisco and Miami to culturally tailor these tools for these priority populations. We then conducted a 1-month technical pilot among 21 participants to assess the usability and acceptability of the adapted interventions and optimize the functionality of these tools.

**Results:**

Participants in focus groups liked the “human touch” of text messages in PrEPmate and thought it would be helpful for scheduling appointments and asking questions. They liked the daily reminder messages, especially the fun facts, gender affirmations, and transgender history topics. Participants recommended changes to tailor the language and messages for Spanish-speaking and transgender populations. For DOT Diary, participants liked the adherence tracking and protection level feedback and thought the calendar functions were easy to use. Based on participant recommendations, we tailored language within the app for Spanish-speaking MSM and transgender women, simplified the sexual diary, and added motivational badges. In the technical pilot of the refined tools, mean System Usability Scale scores were 81.2/100 for PrEPmate and 76.4/100 for DOT Diary (*P*=.48), falling in the “good” to “excellent” range, and mean Client Satisfaction Questionnaire scores were 28.6 and 28.3 for PrEPmate and DOT Diary, respectively (maximum possible score=32). Use of both tools was high over the 1-month pilot (average of 10.5 messages received from each participant for PrEPmate; average of 17.6 times accessing the DOT Diary app), indicating good feasibility for both tools.

**Conclusions:**

Using a user-centered design approach, we culturally tailored PrEPmate and DOT Diary to support daily PrEP use among Spanish-speaking MSM and English- and Spanish-speaking transgender women. Our positive findings in a technical pilot support further testing of these mHealth interventions in an upcoming comparative effectiveness trial.

## Introduction

Preexposure prophylaxis (PrEP) with a daily pill of Truvada (tenofovir disoproxil fumarate/emtricitabine) [1-6] or Descovy (tenofovir alafenamide/emtricitabine) [7] has demonstrated high efficacy for HIV prevention, and these are the most commonly prescribed PrEP regimens in the United States [8]. Although more people are initiating PrEP nationally each year [9], retention in PrEP care has been poor. In clinical practice, 37% to 62% of patients discontinue PrEP by 6 months [10-13], with higher discontinuations among people of color [4,14-16], youths [15,17], and the publicly insured (Medicaid) [18]. Reasons for discontinuation include low self-perceived risk, difficulty taking a daily pill, and cost or insurance lapses [17,19,20]. As many individuals who stop PrEP continue to be exposed to HIV, with a number of seroconversions occurring after PrEP discontinuation [10,13,19,21-23], the lack of PrEP persistence has emerged as one of the critical challenges in PrEP implementation [24]. Strategies to support ongoing PrEP use are critically needed to address HIV disparities and maximize the public health impact of PrEP.

Mobile technologies are increasingly being used to support preventive health behaviors [25-29]. Sexual and gender minority individuals may particularly benefit from mobile health (mHealth) interventions that can increase health-related communication and care access and help address stigma to reduce health disparities. Our team has developed 2 innovative mHealth strategies to support PrEP retention in care and adherence among gay, bisexual, and other men who have sex with men (MSM). First, PrEPmate is an *interactive, bidirectional, text messaging intervention* between PrEP users and their providers that supports PrEP use through its personalized communication component [30]. In 2016, we completed a randomized control trial and demonstrated increased retention in care and PrEP adherence among young MSM in Chicago [31]. Second, DOT Diary is a mobile app that promotes self-management of PrEP use and sexual health through an integrated electronic pill-taking and sex diary that delivers real-time feedback on the level of PrEP protection. The app includes a sexual risk calculator that provides personalized feedback on the overall level of HIV risk on and off PrEP and gamification components to increase app engagement. In 2018, we completed a pilot clinical trial evaluating DOT Diary among young MSM, demonstrating high levels of feasibility, acceptability, and adherence [32].

Although each mHealth approach has demonstrated high acceptability or preliminary impact in clinical trials, these technologies were originally developed for English-speaking MSM. In the United States, Hispanic/Latino people are disproportionately affected by HIV, accounting for 29% of new diagnoses in 2021 [33], and there is currently a gap in access to HIV prevention interventions tailored for Spanish-speaking populations. To prepare for broad-scale implementation, these mHealth tools need to be tailored to Spanish-speaking MSM and English- and Spanish speaking transgender women, 2 populations who are at risk for HIV acquisition and could benefit from additional PrEP support. In this study, we conducted a user-centered design study to elicit preferences to tailor both interventions to support daily PrEP use among Spanish-speaking MSM and English- and Spanish-speaking transgender women in the United States. We then built English and Spanish versions of PrEPmate and DOT Diary into scalable technology platforms and conducted a technical pilot to optimize functionality of both mobile tools. This work was conducted in 2 geographically diverse sites in the United States.

## Methods

### Study Population

Eligible participants were cisgender men or transgender women who were aged 18 years or older, were HIV-negative by self-report, were currently taking PrEP or discontinued PrEP within the past year, owned a smartphone, were willing and able to receive SMS text messages and download the DOT Diary app to their phone (technical pilot only), spoke Spanish (for Spanish focus groups, technical pilot) or English (for English focus groups, technical pilot), and were willing to turn on their video during the focus group (for virtual focus groups).

Participants were recruited across 2 locations/sites: the San Francisco Bay Area in California (Bridge HIV within the San Francisco Department of Public Health) and the greater Miami area in Florida (University of Miami). Recruitment methods included clinic-based recruitment; online and social media strategies (eg, Facebook, Instagram, Craigslist, Grindr, Scruff); distributing posters, flyers, and palm cards advertising the study; and direct outreach at local venues, including community-based organizations and community events. Additionally, we recruited from former study participants who consented to be contacted about future research.

### Interventions

Development and prior testing of PrEPmate and DOT Diary in English-speaking MSM have been described previously [31,34-35]. Briefly, PrEPmate is a text messaging–based intervention grounded in the information, motivation, behavioral skills theory of behavior change [36]. PrEPmate promotes personalized communication between patients and clinic staff through interactive weekly “check-in” messages asking participants how PrEP is going, allowing staff to identify patients needing more help in taking PrEP, and customized daily pill-taking reminder messages [30]. Additionally, the platform provides daily pill-taking reminders in the form of fun facts, affirmations, quotes, and jokes and supports 2-way communication between patients and PrEP navigators, including reminders for upcoming clinic visits. As part of onboarding, participants are provided links to key information about PrEP (PrEP Basics) [37] and video testimonials of peers taking PrEP.

**Figure 1:**
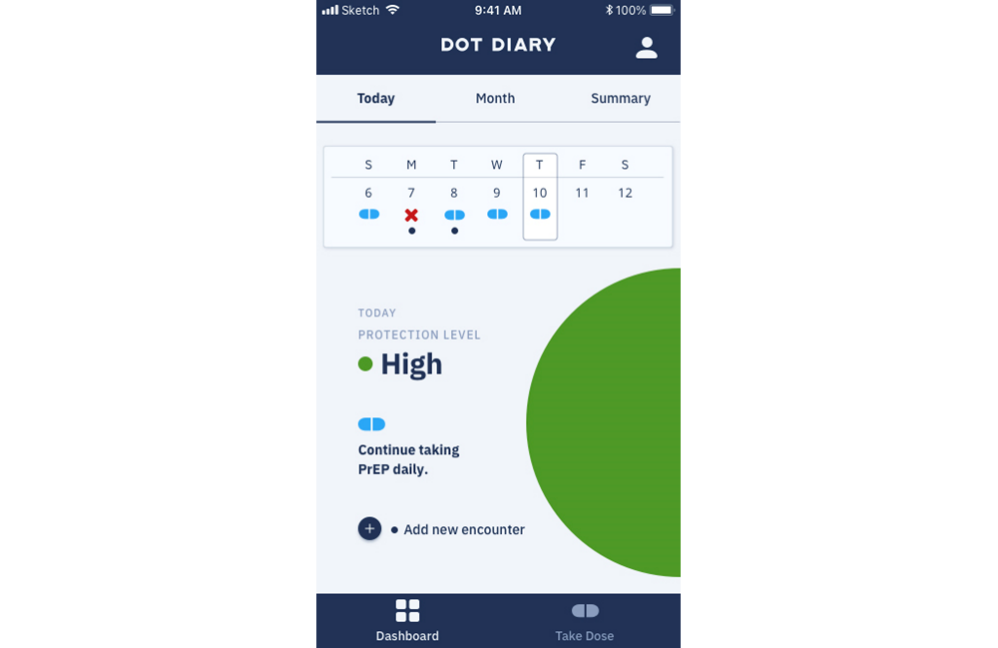
Screenshot of the DOT Diary app.

Dot Diary is a mobile phone app that integrates an electronic pill-taking and sex diary and delivers real-time feedback on PrEP protection. Using the self-management model [38] to increase self-efficacy and patient empowerment, participants log daily PrEP pill-taking and sexual behaviors in the app, which then provides real-time feedback on the level of protection achieved from PrEP (high, medium, low) and customized instructions on doses of PrEP needed to achieve or maintain high protection (Figure 1). Participants can also view a weekly and monthly calendar and a summary page displaying the number of doses in the past 30 days and proportion of sex acts covered by PrEP.

Prior to initiating focus groups, all content in both tools was translated into Spanish through a multistep process. First, a professional translation service was contracted to conduct the initial translation. Our study team provided the translation services with guidelines and examples of language preferred by the community. After receiving the translated text, several native Spanish-speaking research staff reviewed the translations for cultural appropriateness and sensitivity and revised the language to ensure a community-focused perspective. As there are regional differences in Spanish language colloquialism and slang, staff discussed and agreed upon language that could be more broadly acceptable and meet local community standards across different regions. To tailor content for transgender women, additional content was developed and added by research staff, including research staff who identify as transgender.

### Focus Groups for Adapting Interventions

We conducted 3 focus groups with Spanish-speaking MSM and English- and Spanish-speaking transgender women (1 each) in San Francisco and Miami (15 participants total). Eligible participants were consented in person or online using an information sheet prior to initiation of the focus group. Focus groups were conducted using a Health Insurance Portability and Accountability Act–compliant videoconferencing program (Zoom) or in person in a private room. Using a discussion guide, we elicited preferences on intervention content (eg, tailored text messages, dosing reminders), language, layout, usability, and functionality, first for PrEPmate, then for DOT Diary with a focus on tailoring these tools for Spanish-speaking MSM and English- and Spanish-speaking transgender women. Screenshots of the app and text message examples were shared using the share screen function in Zoom. At the end of the group, participants completed a brief postgroup questionnaire on demographics, technology use, and initial impressions of both mHealth PrEP support tools. Focus groups were led by 2 research staff members trained in qualitative methods and user-centered design principles and fluent in English or Spanish (for Spanish focus groups). Groups lasted 90 minutes to 120 minutes and were audio recorded and professionally transcribed and translated (for Spanish focus groups) verbatim.

Guided by user-centered design principles [39], we used rapid qualitative methods to analyze the focus group data [40]. After each focus group, key findings from the discussion were summarized in a debrief note. Data captured in this template were focused on information needed to refine and adapt the interventions for our priority populations. Upon receipt of transcripts from the focus groups, members of the team reviewed the transcripts and captured additional details to ensure completeness. These data were used to refine the app before the initiation of the technical pilot.

### Technical Pilot to Assess Usability and Acceptability of the Adapted Interventions

After finalizing revisions to PrEPmate and DOT Diary, we optimized the functionality of PrEPmate and DOT Diary in a 1-month pilot among 21 MSM and transgender women daily PrEP users recruited in San Francisco and Miami. Eligible participants attended an in-person or online screening visit where they were consented in person or via Docusign and completed a baseline survey to assess demographics, sexual practices, and use of technology. Eligible participants were enrolled, randomized 1:1 to receive either PrEPmate or DOT Diary, and provided access to the intervention. Participants completed in-person or online surveys at baseline and 1-month visits and a brief qualitative exit interview on the acceptability of the intervention at the 1-month visit only. At baseline, participants responded to questions on demographics, sexual behavior, PrEP use, and mobile phone and app use. At the 1-month visit, participants completed the System Usability Scale (SUS) [41], a 10-item instrument that evaluates usability and acceptability of the interventions (scores ranging from 0 to 100), and the Client Satisfaction Questionnaire (CSQ-8) [42], an 8-item instrument that provides scores ranging from 8 to 32. We compared SUS and CSQ-8 scores according to the study arm using *t* tests. Participants were also asked to rate the components of both interventions, complete questions on social benefits and harm, and complete the PrEP Adherence Self-Efficacy Scale (PrEP-ASES) [43]. Participants were reimbursed US $75 for each visit completed. We evaluated app analytics (including logins to the app and use of different app components) for both interventions. Zoom audio recordings of interviews were transcribed verbatim. Interview content was summarized on a debrief template based on the interview guide, and key quotes were also extracted. Additional team members reviewed the transcript of the audio recording and compared it with the debrief report to ensure completeness, and the original interviewer reviewed and validated any changes. Content of the debrief template for each interview was added to a Microsoft Excel file to create a matrix, and rapid qualitative methods were used to analyze interview data [40]. Each participant debrief template was entered in a single row, and each column addressed different domains and feedback on different components of the interventions. Common themes in each column were then synthesized as key findings.

### Ethical Considerations

All study procedures were approved by the University of California San Francisco Institutional Review Board (IRB# 20-30256). Focus group participants were provided an information sheet in English or Spanish describing study procedures and risk and benefits of study participation. Technical pilot participants provided written informed consent prior to initiation of study procedures. Focus group transcripts were transcribed in a way to remove any identifying information. Survey data for both phases only contained de-identified data and were coded by a subject number. A Business Associates Agreement was established with both PrEPmate and DOT Diary developers to ensure confidentiality and security of data. Focus group participants were paid US $75 for completing the focus group, and technical pilot participants were paid US $75 for completing each visit (US $150 total).

## Results

### Findings From the Focus Groups

Demographics of the 15 focus group participants are shown in Table 1. Mean age was 37.9 (SD 9.4) years; 9 (56%) self-identified as transgender woman, and 6 (38%) identified as cisgender men. A substantial proportion self-identified as Latino(a) or Hispanic (6/14, 43%) and reported currently taking PrEP (11/15, 73%), having health insurance (11/15, 73%), having a primary care provider (13/15, 87%), and using iOS (7/15, 47%) or Android (6/15, 40%) mobile phones.

**Table 1:** Sociodemographic characteristics and preliminary acceptability of DOT Diary and PrEPmate among English and Spanish-speaking men who have sex with men and transgender women focus group participants in San Francisco, California and Miami, Florida (N=15).

Characteristic		Results
**Gender, n (%)**		
	Transgender woman	9 (56)
	Cisgender man	6 (38)
Age (years), mean (SD)		37.9 (9.4)
**Age (years), n (%)**		
	18-29	3 (20)
	30-39	5 (33)
	≥40	7 (47)
**Race/ethnicity, n (%)**		
	Latino/a/x or Hispanic	6 (43)
	White	5 (36)
	Black	2 (14)
	Native American	1 (7)
**PrEP^a^ use, n (%)**		
	Prior PrEP use	4 (27)
	Currently taking PrEP	11 (73)
Health insurance (yes), n (%)		11 (73)
Primary care provider (yes), n (%)		13 (87)
**Mobile phone operating system, n (%)**		
	iPhone (iOS)	7 (46)
	Google/Android	6 (40)
	Mobile phone without smartphone features (such as easy internet and email access)	1 (7)
	Nokia smartphone (eg, E62, E71x)	1 (7)
**Mobile phone plan, n (%)**		
	Contract billed monthly	13 (81)
	Shared plan with someone	2 (13)
**Mobile phone plan includes data/internet use, n (%)**		
	Set number of included text messages each month	1 (7)
	Unlimited text messaging	14 (93)
**Mobile phone temporarily disconnected (past 12 months), n (%)**		
	Never	11 (73)
	Once	2 (13)
	Twice	1 (7)
	I don’t know	1 (6.7)
**Mobile phone lost or stolen (past 12 months), n (%)**		
	Never	12 (80)
	Once	1 (7)
	Twice	2 (13.3)
**Would use DOT diary, n (%)**		
	Yes, definitely	9 (60)
	Yes, I think so	5 (33)
	No, I don’t think so	1 (7)
**Would use PrEPmate, n (%)**		
	Yes, definitely	10 (67)
	Yes, I think so	4 (27)
	No, I don’t think so	1 (7)
**If you had a chance, would you prefer to use, n (%)**		
	DOT Diary (mobile app)	5 (33)
	Either PrEPmate or DOT Diary	4 (27)
	Neither PrEPmate nor DOT Diary	4 (27)
	PrEPmate (text messaging program)	2 (13)

^a^PrEP: preexposure prophylaxis.

For PrEPmate, participants liked the “human touch” in the messaging and felt like someone cared about them, although a few participants were unclear if the messages were an automatic response from the system versus a reply from a staff member and said they were unsure if engaging with PrEPmate would lead to communicating with a person. They recommended explaining the messaging system and who would be responding to messages during the onboarding process. They also thought PrEPmate would be helpful for scheduling appointments and answering questions. Participants liked the daily pill-taking reminder messages, especially the fun facts, gender affirmations, transgender historical facts, and jokes, and thought these reminder messages would be most helpful for those who have not yet established their pill-taking routine. In general, transgender women participants liked the tone and language of the trans messages, although they commented that one message related to “two-spirit identity utilized an outdated term” and recommended removing or rephrasing this message. They also recommended adding additional trans-affirming messages and those on the history of transgender people, as well as messages providing information about COVID-19. Spanish-speaking participants suggested text messages in Spanish, such as “Una pastille al dia, te puede salvar la vida” (“one pill a day can save your life”). Regarding frequency of messaging, participants suggested that a text be sent out after an initial 2-week period asking if they would like to continue receiving messages. Participants varied in their message frequency preference, including once a week, twice a week, or once every 2 weeks. Participants felt the links to PrEP facts and brief videos were helpful, particularly the video on how PrEP works in the body that was available in English and Spanish. They recommended additional videos highlighting stories and testimonials from people like them taking PrEP, which could help reduce stigma.

For DOT Diary, participants had generally positive feedback for the app and particularly liked the adherence tracking and protection level features, including the bright colors indicating protection level (red, yellow, green). They also thought the calendar view was clear and understandable and understood what the different symbols represented. Participants felt there were too many questions in the sexual diary and recommended simplifying the information being collected. One participant stated:

...say all I really want out of this, personally, would be like, you know, did I have sex on this date. And then, if it’s relevant to my risk, like maybe what types of acts I engaged in with an option to possibly put a name or more information in a notes field. But like I can see how people would look at this and felt like it was collecting too much information.

In particular, participants expressed concern that entering specific information about a partner and rating them could negatively impact the relationship. Although a participant suggested that it might be helpful if the app could track dosing of other medications, others felt that the app should just stick to reminders and information about PrEP, as adding the tracking of additional medications may overcomplicate the app. Regarding the sexual risk assessment tool component, only a few participants found this would be a helpful tool—possibly those having a larger number of sexual partners with different people on a regular basis. When asked about the sex trends and insights page, some participants felt that it looked too much like a business plan, with too many graphs and being difficult to understand; these participants recommended that the language and appearance be simplified to make it more accessible. Finally, participants appreciated the idea of earning badges:

Yeah. I like the—the way I seem them is I seem the as, you know, achievement, like, for example, Xbox. You do so many things on a particular game, you get an achievement.

This gamification acts as a motivator and affirms that a person is doing well.

Almost all participants reported that they would use PrEPmate (14/15, 93%) and DOT diary (14/15, 93%). For PrEPmate, the daily pill-taking reminder messages were considered the most useful component (13/15, 87%), and the weekly check-in messages were considered both among the most useful (6/15, 40%) and least useful (6/15, 40%) features in PrEPmate. For DOT Diary, the home screen circle indicating level of protection and the ability to track sexual partners and encounters in the diary were considered the most useful components (10/15, 67% for both), and the “badges” were considered the least useful (5/15, 33%).

### Summary of Changes Made to PrEPmate and DOT Diary After Focus Groups

Refinements made to our mHealth tools based on feedback from focus groups are summarized in Table 2. For PrEPmate, we revised our onboarding messaging to clarify that, although the reminder and check-in messages were automated, an actual staff member would respond to messages sent by participants in the system. We also refined the language of trans-specific messages and messages in Spanish based on feedback from participants and added additional content to better tailor the app for these populations. Added examples of transgender affirmative messages and the history of transgender people include:

- “We resist, actively, every time we affirm ourselves.”
- “#StayAffirmed. We got this! Time for our pill of the day.”
- “For generations, South Asian hijra (trans) communities have ‘adopted’ young boys who were rejected by or fled from their biological families.”

We added messages with links to additional video content based on focus group feedback. As most participants appreciated having an initial 2 weeks of reminder messages along with weekly check-in messages, we decided to keep this messaging frequency for the technical pilot.

**Table 2:** Refinements made to PrEPmate and DOT Diary based on feedback from focus groups.

App	Refinements
PrEPmate	Clarified onboarding procedures and who will be responding to messages Refined trans-specific messages and Spanish language messages based on feedback Added links to additional video content
DOT Diary	Reduced number of questions in sexual diary, removed rating feature Removed sexual risk assessment component and simplified sex trends and insights page Added motivational badges

For DOT Diary, participants liked the core features of the app, so we kept the adherence tracking and protection level feedback intact, along with the weekly and monthly calendars summarizing dosing and sexual activity. Based on feedback that the sexual diary was too detailed, we reduced the number of questions and took out the partner rating feature, as several participants found that off-putting. To keep the app streamlined and simple, we decided against adding the ability to track other medications. We also removed the sexual risk assessment tool component of the app given the low level of interest in that feature and simplified the sex trends and insights page based on participant input. Based on participant feedback and input from our Spanish-speaking staff, we revised Spanish-language text in DOT Diary to be more culturally appropriate for sexually active MSM. For example, the phrase for “I rimmed them” was changed from “Le di un beso negro” to “Le comi el culo,” and the phrase for “I jerked them” was changed from “Le hice una paja“ to “Le corri la paja.” Finally, we added a series of badges that participants could earn through use of the app, which could be viewed in a “badge collection” section, based on focus group feedback that gamification could increase engagement and dosing in the app.

### Findings From the Technical Pilot

#### Overview

From July 2021 to August 2021, 21 participants were randomized to the DOT diary (n=11) and PrEPmate (n=10) arms: 12 (57%) in Miami and 9 (43%) in San Francisco. Table 3 describes the baseline characteristics of the technical pilot participants by study arm. Overall, mean age was 38.3 (SD 7.6) years; of the 21 participants, 12 (57%) self-identified as cisgender man, and 9 (43%) identified as transgender women; and 9 (43%) of the 21 participants tested the tools in Spanish. The majority self-identified as Latino(a) or Hispanic (17/21, 81%), had completed a college education or higher (12/19, 63%), were currently employed (13/20, 65%), lived in their own house or rented a house (17/21, 81%), and did not have a primary partner (15/20, 71%). The mean number of partners was 2.0 (SD 3.1) in the past 3 months; approximately one-half reported anal sex (10/21, 48%), use of alcohol (11/21, 52%), and any substance use (13/21, 62%) in the past 3 months. A total of 12 (12/21, 57%) participants were using PrEP for more than 12 months, and 15 (15/21, 71%) reported a very good or excellent ability to take daily PrEP during the study. Most participants (15/21, 71%) reported using the iOS mobile phone operating system, and 6 (6/21, 29%) reporting using an Android phone. Most participants had a phone contract billed monthly (15/19, 79%) and used 5 or more apps weekly (15/21, 75%). Of the 21 participants, 15 (71%) reported using apps for dating, 13 (62%) reported using apps for health, and 12 (57%) reported using apps to send reminders.

**Table 3:** Baseline characteristics of the technical pilot participants in San Francisco, California and Miami, Florida by study arm (DOT Diary or PrEPmate).

Characteristics		DOT Diary (n=11)	PrEPmate (n=10)	Total sample
**Site, n (%)**				
	San Francisco	5 (46)	4 (40)	9 (43)
	Miami	6 (55)	6 (60)	12 (57)
**Language, n (%)**				
	English	7 (64)	2 (20)	9 (43)
	Spanish	4 (36)	8 (80)	12 (57)
Age (years), mean (SD)		37.3 (9)	39.5 (7)	38.3 (8)
**Age (years), n (%)**				
	18-29	3 (27)	1 (10)	4 (19)
	30-39	2 (18)	5 (50)	7 (33)
	≥40	6 (55)	4 (40)	10 (48)
**Gender, n (%)**				
	Cisgender man	6 (55)	6 (60)	12 (57)
	Transgender woman	5 (46)^a^	4 (40)	9 (43)
**Sexual orientation, n (%)**				
	Gay	7 (64)	5 (50)	12 (57)
	Queer	1 (9)	3 (30)	4 (19)
	Bisexual	2 (18)	2 (20)	4 (19)
	Straight	1 (9)	0	1 (5)
**Race/ethnicity, n (%)**				
	Latino(a) or Hispanic	8 (73)	9 (90)	17 (81)
	Black	1 (9)	0	1 (5)
	Asian^b^	0	1 (10)	1 (5)
	White	1 (9)	0	1 (5)
	Native American	1 (9)	0	1 (5)
**Education, n (%)**				
	Completed college or higher	6 (60)	6 (67)	12 (63)
	Less than college	4 (40)	3 (33)	7 (37)
**Annual income (US $), n (%)**				
	0-29,999	6 (55)	2 (20)	8 (38)
	≥30,000	0	5 (50)	5 (24)
	Not declared	5 (46)	3 (30)	8 (38)
**Employment, n (%)**				
	Employed	6 (60)	7 (70)	13 (65)
	Full-time student	1 (10)	0	1 (5)
	Unemployed	2 (20)	2 (20)	4 (20)
	Disabled	1 (10)	1 (10)	2 (10)
**Housing, n (%)**				
	Living in someone’s house	2 (18)	1 (10)	3 (14)
	Living in own/rent house	9 (82)	8 (80)	17 (81)
	Living in hotel	0	1 (10)	1 (5)
Live alone (yes), n (%)		4 (36)	3 (30)	7 (33)
Health insurance (yes), n (%)		6 (60)	7 (70)	13 (65)
Primary care provider (yes), n (%)		5 (50)	7 (70)	12 (60)
**Primary partner, n (%)**				
	No	10 (91)	5 (50)	15 (71)
	Yes	0	4 (40)	4 (19)
	Not sure	1 (9)	1 (10)	2 (10)
Number of partners^c^, mean (SD)		2.9 (3.9)	1.1 (1.9)	2.0 (3.1)
Anal sex^c^, n (%)		5 (46)	5 (50)	10 (48)
Alcohol use^c^, n (%)		6 (45)	5 (50)	11 (52)
Any substance use^c^, n (%)		7 (64)	6 (60)	13 (62)
**Time on PrEP^d^ (months), n (%)**				
	<6	3 (27)	3 (30)	6 (29)
	6-12	1 (9)	2 (20)	3 (14)
	>12	7 (64)	5 (50)	12 (57)
**Ability to take daily PrEP during the study^e^, n (%)**				
	Very poor or fair	1 (9)	1 (10)	2 (10)
	Good	1 (9)	3 (30)	4 (19)
	Very good	5 (46)	3 (30)	8 (38)
	Excellent	4 (36)	3 (30)	7 (33)
**Mobile phone operation system, n (%)**				
	Google/Android	1 (9)	5 (50)	6 (29)
	iPhone (iOS)	10 (91)	5 (50)	15 (71)
**Mobile phone plan, n (%)**				
	Contract billed monthly	7 (78)	8 (80)	15 (79)
	Prepaid	1 (11)	1 (10)	2 (11)
	Shared plan with someone	1 (11)	1 (10)	2 (11)
**Mobile phone temporarily disconnected^f^, n (%)**				
	Never	10 (91)	7 (78)	17 (85)
	Once or twice	1 (9)	2 (22)	3 (15)
**Mobile phone lost, stolen, or broken^f^, n (%)**				
	Never	8 (73)	5 (56)	13 (65)
	Once or twice	3 (27)	4 (44)	7 (35)
**Type of app used, n (%)**				
	Dating (yes)	10 (91)	5 (50)	15 (71)
	Health (yes)	7 (64)	6 (60)	13 (62)
	Send reminders (yes)	6 (55)	6 (60)	12 (57)

^a^One participant also identified as gender queer.

^b^Participant also identified as Native American.

^c^Past 3 months.

^d^PrEP: preexposure prophylaxis.

^e^Assessed at baseline.

^f^In the past 12 months.

Mean SUS scores were 81.2 (SD 13.1) for PrEPmate and 76.4 (SD 12.2) for DOT diary (*P*=.48), corresponding to adjective ratings in the “good” to “excellent” range [44] (Table 4). The highest score for both mHealth tools was on the item “I thought the system was easy to use” (DOT Diary: 4.9, SD 0.4; PrEPmate: 4.8, SD 0.5). The score on the item “I found the various functions in DOT Diary/PrEPmate were well integrated” was higher for PrEPmate than for DOT diary (4.6, SD 0.7 vs 3.6, 1.4; *P*=.09).

**Table 4:** Mean System Usability Scale (SUS) and Client Satisfaction Questionnaire (CSQ-8) scores among the technical pilot participants in San Francisco and Miami by study arm (DOT Diary or PrEPmate).

Questionnaire responses			DOT Diary, mean (SD)		PrEPmate, mean (SD)		Total sample, mean (SD)		*P* value^a^
**SUS^b^**									
	1. I think that I would like to use DOT Diary/PrEPmate on a regular basis.	4.7 (0.5)		4.5 (0.8)		4.6 (0.6)		.53	
	2. I found DOT Diary/PrEPmate unnecessarily complex.	2.7 (1.7)		2.4 (1.7)		2.5 (1.6)		.70	
	3. I thought the system was easy to use.	4.9 (0.4)		4.8 (0.5)		4.8 (0.4)		.63	
	4. I think that I would need the support of a technical person to be able to use DOT Diary/PrEPmate.	2.4 (1.5)		2.4 (1.7)		2.4 (1.5)		.95	
	5. I found the various functions in DOT Diary/PrEPmate were well integrated.	3.6 (1.4)		4.6 (0.7)		4.1 (1.2)		.09	
	6. I thought there was too much inconsistency between different parts of DOT Diary/PrEPmate.	2.3 (1.1)		1.5 (1.1)		1.9 (1.1)		.19	
	7. I would imagine that most people would learn to use DOT Diary/PrEPmate very quickly.	4.7 (0.8)		4.6 (0.7)		4.7 (0.7)		.82	
	8. I found DOT Diary/PrEPmate very cumbersome to use.	2.7 (1.4)		1.6 (1.1)		2.1 (1.3)		.11	
	9. I felt very confident using DOT Diary/PrEPmate.	4.9 (0.4)		4.2 (1.4)		4.5 (1.1)		.28	
	10. I needed to learn a lot of things before I could get going with DOT Diary/PrEPmate.	2.0 (1.4)		2.4 (1.5)		2.2 (1.4)		.63	
	Total score (0-100)	76.4 (12.2)		81.2 (13.1)		79.0 (12.5)		.48	
**CSQ-8^c^**									
	How would you rate the quality of DOT Diary/PrEPmate?	3.6 (0.8)		3.6 (0.5)		3.6 (0.6)		.88	
	Did you get the kind of support you wanted from DOT Diary/PrEPmate?	2.9 (1.1)		3.4 (0.9)		3.1 (1.0)		.33	
	To what extent did DOT Diary/PrEPmate meet your needs for PrEP support?	3.6 (0.5)		3.5 (0.8)		3.5 (0.6)		.84	
	Would you recommend DOT Diary/PrEPmate to a friend?	3.7 (0.5)		3.6 (0.5)		3.7 (0.5)		.74	
	How satisfied are you with the amount of help (DOT Diary/PrEPmate) you received from DOT Diary/PrEPmate?	3.7 (0.5)		3.4 (1.1)		3.5 (0.8)		.45	
	Did using DOT Diary/PrEPmate help you take PrEP daily?	3.6 (0.5)		3.8 (0.5)		3.7 (0.5)		.50	
	In an overall, general sense, how satisfied are you with DOT Diary/PrEPmate?	3.7 (0.5)		3.8 (0.5)		3.7 (0.5)		.89	
	If you enrolled in the future in a clinical trial to help researchers discover and evaluate treatments, would you use DOT Diary/PrEPmate?	3.6 (0.5)		3.6 (0.5)		3.6 (0.5)		.85	
	Total score (8-32)	28.3 (3.2)		28.6 (4.4)		28.5 (3.8)		.87	

^a^*t* test.

^b^Scores range from 1 (strongly disagree) to 5 (strongly agree).

^c^Scores range from 1 to 4.

Mean CSQ-8 scores were 28.6 (SD 4.4) for PrEPmate and 28.3 (SD 3.2) for DOT Diary (*P*=.87). The highest scores (3.7) were on items “Would you recommend DOT Diary/PrEPmate to a friend?”, “Did using DOT Diary/PrEPmate help you take PrEP daily?”, and “In an overall, general sense, how satisfied are you with DOT Diary/PrEPmate?” The lowest score (3.1) was on item “Did you get the kind of support you wanted from DOT Diary/PrEPmate?”

**Figure 2:**
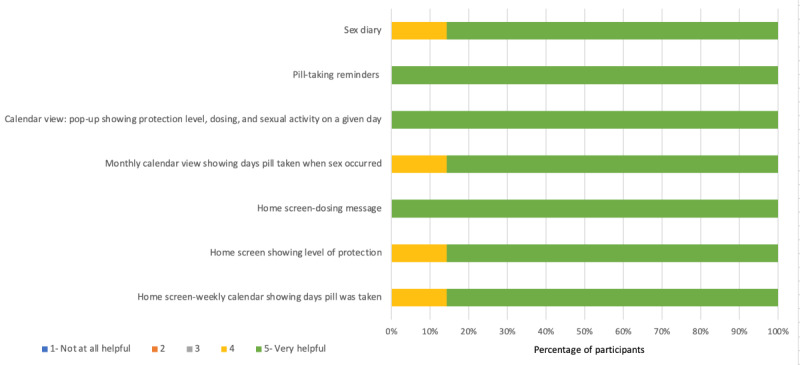
Ratings of the components of the DOT Diary among the technical pilot participants in San Francisco and Miami.

**Figure 3:**
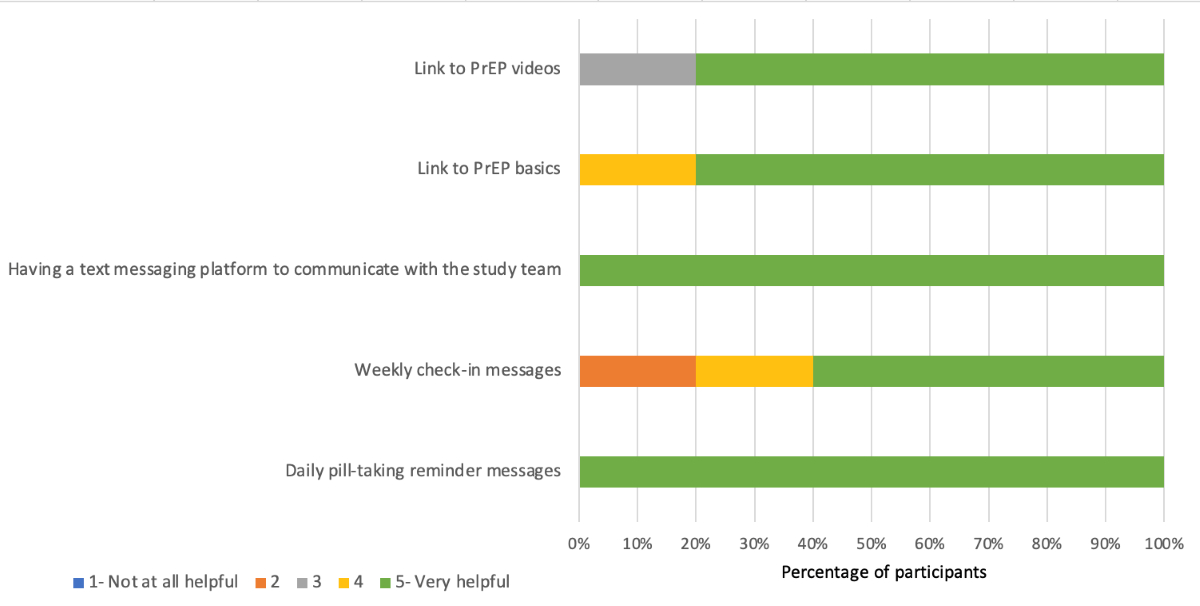
Ratings of the components of PrEPmate among the technical pilot participants in San Francisco and Miami. PrEP: preexposure prophylaxis.

Most of the participants (60-100%) rated each of the components of DOT Diary and PrEPmate as a 5 (very helpful) on a 5-point Likert scale (Figures 2 and 3). For DOT Diary, all participants rated the components “Daily reminder notification to take pill,” “Calendar view: pop-up showing protection level, dosing, and sexual activity on a given day,” and “Home screen indicating level of protection” as very helpful. For PrEPmate, all participants rated the components “Having a text messaging platform to communicate with the study team” and “Daily pill-taking reminder messages” as very helpful. Most participants reported they reviewed the PrEP Basics information (6/8, 75%), and one-half reported they watched the PrEP videos (4/8, 50%).

At the 1-month follow-up visit, the mean PrEP-ASES score was 9.4 (SD 1.6) for DOT Diary and 9.3 (SD 0.9) for PrEPmate (*P*=.80).

After participating in this study, most participants reported feeling good about helping others or the community (15/16, 94%), being more confident in taking PrEP (14/16, 88%), and having a better understanding of sexual behaviors and risk (14/16, 88%). One participant reported a social harm related to having trouble getting or keeping housing in the DOT diary arm.

#### Feasibility of the Tools

Among 10 participants assigned to the PrEPmate arm, 1 participant withdrew at the enrollment visit, and the remaining 9 participants responded to at least one check-in message. During their study participation, the mean total number of incoming messages sent from participants was 10.5 (range 4 to 42), and the mean time to the first response for each weekly check-in message was 21 hours (range 13 minutes to 67 hours). Most incoming messages from participants were in response to weekly check-ins, and some were related to scheduling appointments.

Among 11 participants assigned to the DOT Diary arm, they opened the app, on average, 17.6 (SD 21.4; range: 2-67) times, with an average duration of 2.77 (SD 3.12) minutes per session (range: 5 seconds to 9.49 minutes). Across all participants, the cumulative time spent in the app ranged from 15 seconds to 2 hours and 26.5 minutes, with an average of 34 (SD 43.8) minutes. The most frequently used functions included diary entries (mean 79.9, SD 132.2 accesses) and taking a dose (15.9, SD 21.2 accesses), whereas the least used features were the monthly calendar view (mean 3.7, SD 4.5 accesses) and the summary statistics page (mean 1.3, SD 2.1 accesses).

#### Exit Interviews

The follow-up exit interviews were completed by 17 participants approximately 1 month after enrollment (10 in the DOT Diary arm, 7 in the PrEPmate arm). Regarding PrEPmate, most participants found the system straightforward and easy to use and navigate. They found the onboarding process to be clear and fast, and the onboarding links were a good resource for PrEP information. Both Spanish-speaking and transgender participants reported liking the daily fun facts, affirmations, historical messages, and jokes, which were interesting and not too intrusive. The regularity of these daily messages was helpful as pill-taking reminders, as a Spanish-speaking participant said, “A very – very – very accurate reminder,” and was particularly helpful on days when participants were out of their usual routine, such as waking up late or if they were on their day off. Additionally, participants found access to a PrEP navigator helpful, as it provided reminders to get labs completed. As constructive feedback, participants said that more variety and types of messages were needed, including other topics not related to LGBTQIA+ communities, as over time, the messages “start[ed] to get a little stale,” remarked an English-speaking transgender participant. Participants suggested having more information about PrEP efficacy and the window of time they had to take their pill, as well as HIV testing and other HIV prevention strategies. Participants liked the weekly interaction and being checked on by the navigator, although some participants were still unclear whether messages were automated or coming from an actual person and suggested that this be clarified during the onboarding process, with the staff member saying, “I will occasionally reach out to you through PrEPmate.”

Regarding DOT Diary, participants appreciated logging their encounters and receiving notifications, and the reminders for taking daily PrEP were especially useful, particularly for those who don’t have an established pill-taking routine. Participants also found it helpful to know their protection levels, which made them feel more confident having sexual encounters; they also liked the feature showing how many more doses were needed to reach full protection levels. Reported cons of DOT Diary included not being able to retroactively log a dose taken earlier, not seeing the little dot added to the calendar that identified an encounter, and not having reminders for an upcoming 3-month lab visit. Several participants commented on the language used in the app. A young transgender woman (age 24 years) in San Francisco felt the pop-up congratulatory message after taking a dose was a bit “cheesy,” saying “it felt like it was someone who was, like 50, trying to relate to me as a youth, which always feels weird.” A 46-year-old Spanish-speaking MSM in Miami was shocked and amused by the Spanish translation of language in the app and felt that the language was sexually empowering but also a bit taboo, while a 41-year-old English-speaking transgender woman in San Francisco found the language used to identify sex acts as “grotesque.” Several participants liked reporting their sexual encounters, saying “it’s a calendar of your sex life,” and liked seeing a visual record of their sexual encounters, as it helped them be more mindful of their sexual activity:

It’s like a mirror right? Because it perhaps shows you in another way what your activity has been or Okay, I see that perhaps I’m more active on the weekends or anything. So, it gives you like that reflection of what our activity is.

An MSM in Miami reported the amount of information the app requested was somewhat intrusive, although he said this was not a dealbreaker and may have found it more useful if he had more sexual partners. Several participants recommended including a function to add doses retroactively and to support on-demand or 2-1-1 PrEP dosing. Participants also wanted to be able to customize the alarm ring tone, as some of the alarm tones were low volume and difficult to hear. On the summary data page, one participant suggested adding information about the total number of sexual partners for the month. Finally, most participants found the onboarding experience easy, although 1 participant found the onboarding survey a bit tedious and another participant recommended separating questions about their male and transfemale partners.

## Discussion

### Principal Findings

This study describes findings from 2 phases of formative research, including focus groups and a technical pilot, to tailor 2 innovative mHealth daily PrEP support tools for Spanish-speaking MSM and English- and Spanish-speaking transgender women in the United States. In addition to translating the tools into Spanish, key adaptations informed by our formative work included culturally tailoring content and language within our interventions to our 2 priority populations (both PrEPmate and DOT Diary), clarifying that navigators will respond to text messages from participants during the onboarding process (PrEPmate), and simplifying the sexual diary and adding motivational badges to the app (DOT Diary). In a 1-month technical pilot of these tools, both PrEPmate and DOT Diary demonstrated feasibility based on use of the tools as assessed via paradata and good-to-excellent acceptability assessed via self-report.

A growing number of mHealth tools are being designed and evaluated to support PrEP uptake, adherence, and persistence [45]; however, few tools have been specifically designed or tailored for Spanish-dominant individuals in the United States. Several studies suggest that culturally adapted behavioral interventions for Latino men may outperform standard treatment, and culturally and linguistically tailored resources in Spanish to support PrEP use among MSM and transgender women could help address disparities in PrEP use in these populations [46]. Recent studies have highlighted community-centered approaches to developing these tools for Spanish-speaking populations. MacCarthy and colleagues [47] investigated strategies for improving mobile technology–based HIV prevention interventions with Latino MSM and Latina transgender women. Key findings included that requiring smartphone use could reduce participation in low-income participants; variability in participant preferences regarding personalization, frequency, and timing of text messages; recommendation for messages to be sent on the same days and at the same times to help participants anticipate receiving information; and the importance of recognizing different language literacies and diverse countries of origin. Although the PrEPmate text-messaging intervention can be used on non-smartphones, the DOT Diary intervention does require use of a smartphone. Tracking individuals who were excluded due to not having a smartphone will be important in future studies and implementation to assess the impact of this requirement on equitable outcomes. Regarding message timing, in both PrEPmate and DOT Diary, the timing of messages can be customized and are set to be delivered at the same time each day. Cantos and colleagues [48] used the Assessment, Decision, Adaptation, Production, Topical Experts-Integration, Training, and Testing (ADAPT-ITT) framework to develop an HIV prevention mobile app to increase uptake of PrEP among Latino sexual minority men called SaludFindr. Through in-depth interviews, they found both general barriers that were common to non-Latino groups, as well as Latino-specific barriers, including lack of available clinics to provide culturally concordant care and limited availability of Spanish language information on PrEP.

Although most technology-based HIV prevention tools being developed have focused on MSM, a few mHealth interventions have been tailored specifically for transgender women. In a cross-disciplinary scoping review of mobile technology interventions to improve HIV prevention and care in transgender and gender diverse youth, Skeen and Cain [49] highlighted the importance of gender affirmation as a key social determinant of health for transgender youth, and several interventions have been guided by the gender-affirmative framework [50], which centers the interactive process in which a person receives social recognition and support for their gender identity and expression. In this review, behavioral self-monitoring and access to HIV prevention services were the most frequent features across disciplines. Similarly, our DOT Diary app utilizes self-monitoring and management to promote building of a daily pill-taking routine, and content within our interventions is designed to support gender affirmation (eg, trans-specific affirmation messages in PrEPmate). In the TechStep study, Reback and colleagues [51] developed several culturally responsive technology-based interventions (text messaging, web app, and eCoaching) tailored for transgender and gender diverse youth through focus groups and consultation with youth advisory boards in the Adolescent Trials Network, which was subsequently evaluated in a 3-arm sequentially randomized HIV prevention trial. Finally, Morris and colleagues [52] tailored the individualized Texting for Adherence Building (iTAB) intervention through focus groups with transgender and nonbinary individuals and evaluated this approach alone and in combination with motivational interviewing in a large randomized controlled trial among transgender and nonbinary PrEP users. In this study, adherence outcomes as measured by drug levels were mostly similar between the arms, although self-reported adherence was higher in the iTAB + motivational interviewing group versus the iTAB alone group. Together, these studies highlight the importance of adopting a user-centered approach to tailoring mHealth interventions for our priority populations.

The next step for our mHealth tools will be to incorporate changes suggested in our technical pilot exit interviews to further tailor these tools in Spanish and for transgender women. For PrEPmate, we will refine the content and language used in the text messages based on participant feedback and augment the onboarding process to have navigators clearly describe their role in monitoring and responding to text messages. For DOT Diary, we will add a function to retroactively add a dose after the dosing period has passed and refine the language used in the app based on participant feedback. Additionally, through another study, we will adapt and tailor this app to support on-demand and 2-1-1 PrEP use among MSM (1R34MH121139). With both of these tools optimized, we will then conduct a large multisite randomized controlled trial to compare the effectiveness of PrEPmate versus DOT Diary among English- and Spanish-speaking MSM and transgender women through a study funded by the Patient-Centered Outcomes Research Institute.

### Limitations

This study had several limitations. First, the number of participants enrolled in the focus groups and technical pilot was small; therefore, the pilot study was not designed to evaluate the efficacy of the interventions. In addition, due to the short duration of the pilot, longer-term feasibility and acceptability could not be evaluated, and participants were only able to test 1 of the 2 interventions. Still, formative work through focus groups and optimizing usability in a technical pilot are important steps in developing and tailoring interventions prior to evaluating their efficacy in large randomized controlled trials. Social desirability may have impacted participant responses in the focus groups and technical pilot; however, we used a computer-assisted self-interview to assess usability and acceptability, we used paradata metrics from PrEPmate and DOT Diary to objectively assess feasibility, and participants in focus groups and exit interviews were reminded that there were no right or wrong answers and were encouraged to provide honest feedback that would help us best improve the tools. Additionally, for both phases of this study, participants were enrolled in San Francisco and Miami, potentially limiting generalizability of the findings. However, both regions are in high-priority Ending the HIV Epidemic jurisdictions and have large Spanish-speaking Latinx populations, and one site is located in the South, the region accounting for over one-half of new HIV diagnoses nationally. In addition, Miami-Dade County is the metropolitan statistical area with the highest rate of new HIV diagnoses in the United States [53]. Despite these limitations, our study had several strengths, including enrolling a substantial proportion of Spanish-dominant participants and transgender women across 2 diverse sites; the user-centered approach incorporating input from our participants into the final design of our interventions; and the mixed methods approach to evaluate feasibility and acceptability in our technical pilot.

### Conclusions

Using a user-centered design approach, we tailored the PrEPmate text messaging intervention and the DOT Diary mobile app to support PrEP adherence and persistence for Spanish-speaking MSM and English- and Spanish-speaking transgender women in the United States. Preliminary testing in a technical pilot demonstrated high acceptability and feasibility of the adapted versions of these tools and support further evaluation in an upcoming comparative effectiveness trial of PrEPmate versus DOT Diary in a large national US study.

## Abbreviations

ADAPT-ITT: Assessment, Decision, Adaptation, Production, Topical Experts-Integration, Training, and Testing
CSQ-8: Client Satisfaction Questionnaire
iTAB: individualized Texting for Adherence Building
mHealth: mobile health
MSM: men who have sex with men
PrEP: preexposure prophylaxis
PrEP-ASES: PrEP Adherence Self-Efficacy Scale
SUS: System Usability Scale

## Data Availability

Survey data for the technical pilot have been uploaded to the Dryad Repository [54]. Focus group transcripts will be made available upon request.
